# ROS‐responsive robust hydrogels based on thiol‐ene click chemistry for enhancing the healing of infected wounds

**DOI:** 10.1002/btm2.70143

**Published:** 2026-04-08

**Authors:** Huaping Li, Jinbao Zhong, Chao Bi, Bihua Liang, Shanshan Ou, Jiaoquan Chen, Luoyu Zhang, Hui Zou, Tianyi Lin, Sanquan Zhang, Huilan Zhu

**Affiliations:** ^1^ Guangzhou Dermatology Hospital. Institute of Dermatology Guangzhou Medical University Guangzhou People's Republic of China

**Keywords:** antibacterial activity, infected wound repair, ROS response, thiol‐ene click reaction

## Abstract

The development of novel antimicrobial wound healing dressings has become an urgent challenge with the increasing prevalence of drug‐resistant bacterial infections. Herein, a reactive oxygen species (ROS) responsive robust hydrogel (THMA/TCMC‐Gel@Cur) based on “thiol‐ene” click chemistry was developed for infected wounds. The addition of curcumin‐loaded gelatin nanoparticles (Gel@Cur) imparted excellent antibacterial and antioxidant properties to the THMA/TCMC‐Gel@Cur hydrogel. Thiols and alkenes quickly formed thioether bonds through the “thiol‐ene” click chemical reaction. The hydrophobic thioether bonds could be oxidized to hydrophilic sulfoxide bonds by ROS. THMA/TCMC‐Gel@Cur hydrogel formed by “thiol‐ene” click chemistry could respond to ROS and increase water molecules to enter the interior of the hydrogel by switching the hydrophilic to hydrophobic characteristics, thereby accelerating drug diffusion in the porous network. In vivo results showed that wounds treated with THMA/TCMC‐Gel@Cur hydrogel had a wound closure ratio of 93.56% within 14 days, which was significantly higher than the other treatment groups. The THMA/TCMC‐Gel@Cur hydrogel exhibited significant potential for clinical application, poised to enhance the quality of life for patients suffering from infectious dermatological disorders.

## Graphical abstract

A ROS‐responsive hydrogel (THMA/TCMC‐Gel@Cur) based on “thiol‐ene” click chemistry changes hydrophilic characteristics and accelerates drug diffusion, while removing excess ROS while killing bacteria. THMA/TCMC‐Gel@Cur hydrogel is poised to treat infectious wounds.

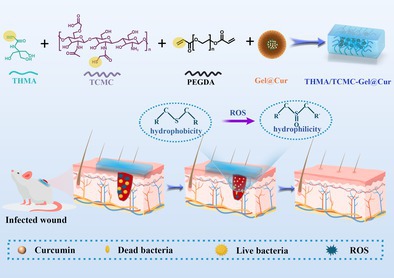

**Translational Impact Statement**

Bacterial infection poses a key challenge in wound healing. The application of antibiotic‐loaded hydrogel has a risk of inducing increased bacterial resistance. We presented a ROS‐responsive robust hydrogel (THMA/TCMC‐Gel@Cur) based on “thiol‐ene” click chemistry for infected wounds. THMA/TCMC‐Gel@Cur could switch the hydrophobic to hydrophilic characteristics by responding to ROS, controlling drug release and killing bacteria. THMA/TCMC‐Gel@Cur could also significantly promote infected wound healing by reducing inflammation, promoting new blood vessel regeneration, and collagen deposition. These results suggested the possibility of THMA/TCMC‐Gel@Cur as more effective healing solutions, enhancing the quality of life for patients suffering from infectious wounds.

## 1 INTRODUCTION

As the largest organ and the initial barrier of the human body, the skin is readily susceptible to diverse forms of injury.^1^, ^2^, ^3^ When skin wounds manifest, the secretion of reactive oxygen species (ROS) by immune cells serves to inhibit bacterial penetration and avert the onset of infection.^4^ However, long‐term bacterial infection induces the overexpression of ROS in the wound, which damages endothelial cells and blood vessels and prolongs the inflammatory phase, hindering the healing of the infected wound.^5^ It is imperative to advance wound dressings that are capable of simultaneously eliminating excess ROS and eradicating bacteria.

Over the past few years, a wide range of new wound dressings have been created to help promote wound healing, such as thin films, aerogel sponges, and hydrogels. Among these dressings, hydrogels have become the most promising wound dressing materials due to their excellent hydrophilicity, good biocompatibility, and three‐dimensional porous structure similar to extracellular matrix.^6^, ^7^, ^8^ In particular, the ROS‐responsive hydrogel can respond to the ROS in the wound microenvironment and release drugs to inhibit bacterial growth and promote wound repair.^9^, ^10^, ^11^ Borate ester bond‐mediated hydrogels represent a common class of ROS‐responsive hydrogels, wherein the dynamic boronic ester bonds undergo cleavage in the presence of ROS, thereby facilitating the rapid release of pharmaceutically encapsulated agents within the hydrogel network.^12^ Although the ROS‐responsive properties of borate ester bond‐based hydrogels have advantages in controlled drug release, the preparation process often requires multiple steps of complex chemical modification, which makes it difficult to be widely used in practical clinical treatment. The thioether hydrogels formed by “thiol‐ene” click chemistry are not only ROS‐responsive, but also simpler and faster to prepare than the borate‐based hydrogels.^13^ The formation of thioether‐based hydrogels proceeds through thiol‐ene click chemistry, as a thiol radical rapidly adds to an olefin double bond via radical addition, forming the thioether bond.^14^ Thioether bonds with hydrophobic properties are able to be oxidized to hydrophilic sulfoxide bonds by ROS. Therefore, ROS oxidizes the hydrophobic thioether to hydrophilic sulfoxide. This change in hydrophilicity allows water molecules to enter the interior of the hydrogel, thereby accelerating the drug diffusion. In the face of the treatment challenge of infectious wounds, the correct choice of drugs is directly related to the success or failure of treatment. Antibiotics are commonly used to treat infected wounds in clinical practice, but the excessive use of antibiotics can exacerbate the development of bacterial resistance, resulting in many common infections becoming difficult to treat. Therefore, a new antimicrobial agent is urgently needed to deal with the harm caused by bacterial infection. Curcumin (Cur) is a natural plant‐derived antibacterial agent. Its molecular structure and antioxidant properties have exhibited significant impacts in inhibiting bacterial growth and reducing ROS accumulation.^15^ In contrast to traditional antibiotic drugs, the utilization of Cur as an antibacterial agent can mitigate the development of bacterial resistance while alleviating oxidative stress caused by elevated ROS levels among cells. However, the poor water solubility and rapid metabolism of Cur in vivo make it difficult to maintain an effective therapeutic level at the wound site, hindering the widespread application of Cur in wound treatment.^16^ Nanotechnology provides a solution to this problem, especially biomass nanoparticles, such as gelatin nanoparticles, which can effectively increase the solubility and bioavailability of drugs through their unique size and surface effects, prolonging the residence time of drugs in the wound.^17^, ^18^ But gelatin nanoparticles will easily agglomerate due to high surface energy and lose their unique properties. Loading gelatin nanoparticles into hydrogels can effectively solve this problem, and the 3D network structure of the hydrogel can uniformly disperse the gelatin nanoparticles and avoid agglomeration. At the same time, it can enhance the controlled drug release of gelatin nanoparticles to achieve sustained drug release and on‐demand drug release.

**FIGURE 1:**
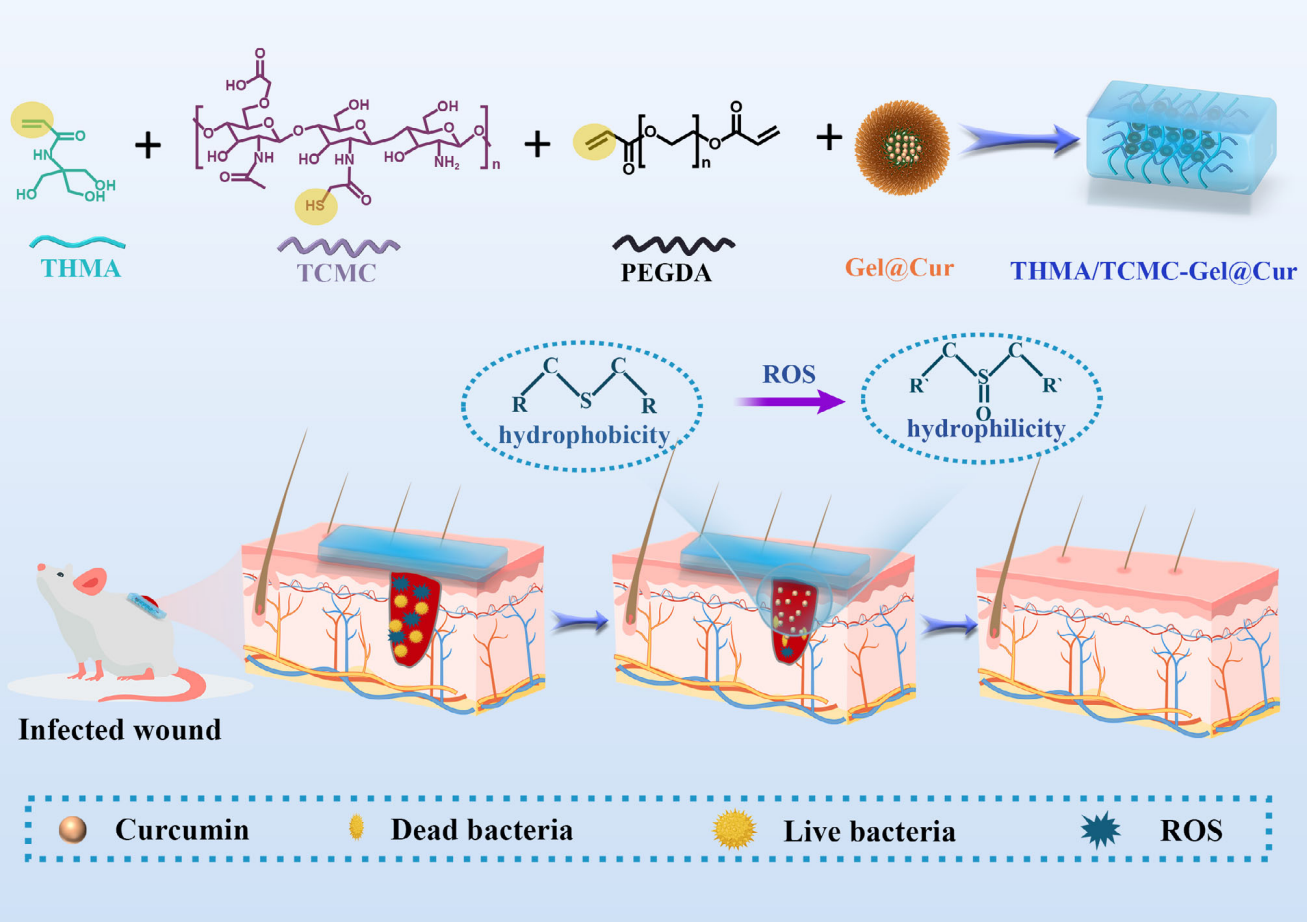
Preparation of THMA/TCMC‐Gel@Cur hydrogel for wound healing purposes.

This study presented a ROS‐responsive hydrogel (THMA/TCMC‐Gel@Cur) based on “thiol‐ene” click chemistry that was used to promote the healing of infected wounds (Figure 1). N‐ [tris (hydroxymethyl) methyl] acrylamide (THMA) and thiolated carboxymethyl chitosan (TCMC) were used as the main materials, and thioether bonds were formed through the “thiol‐ene” click chemical reaction, which enabled the composite hydrogel to respond to ROS. The thioether bond was sensitive to ROS and could be oxidized into more hydrophilic sulfoxide (–SO–) or sulfone (–SO_2_–) structures. The incorporation of Cur‐coated gelatin nanoparticles (Gel@Cur) resulted in the development of a composite hydrogel with remarkable antibacterial and antioxidant capabilities. The THMA/TCMC‐Gel@Cur hydrogels can accelerate the healing of infected wounds by releasing Cur on demand according to the level of ROS in the wound. This composite hydrogel has a good clinical application prospect and is expected to improve the condition of patients with infectious skin diseases, thereby improving their quality of life.

## 2 MATERIALS AND METHODS

### 2.1 Materials

The N‐(1,3‐dihydroxy‐2‐(hydroxymethyl) propan‐2‐yl) acrylamide (THMA, *M*n = 175.18, 97%), gelatin, Cur, Dimethylaminopropyl‐3‐ethylcarbodiimide (EDC, *M*n = 152.23, 99.7%), polyethylene glycol diacrylate (PEGDA, *M*n = 400, 99.7%) and photoinitiator I2959 were provided by Shanghai Macklin company. TCMC was obtained through the modification of carboxymethyl chitosan (CMC, *M*n = 9000 Da, Macklin, Shanghai) with thioglycolic acid (TGA, Kermel, Tianjin). All other reagents were employed without further purification.

### 2.2 Preparation of THMA/TCMC‐Gel@Cur hydrogel

First, Gel@Cur nanoparticles were synthesized according to previously reported methods.^16^ Gelatin (0.5 g) was mixed with 50 mL ultrapure water for 30 min. The mixture was then warmed to 45°C and stirred to dissolve the solid. Adjust pH to 12.0 with NaOH, add 0.1 g Cur powder (271 μmol), and stir continuously for 2 h. To obtain a suspension of Gel@Cur, the pH of the mixture was balanced to 7.0 with acetic acid. Supernatant was recovered by centrifugation for 30 min at 3000 rpm. THMA was homogeneously mixed with TCMC and Gel@Cur nanoparticles and PEGDA and I2959 to obtain the hydrogel precursor mixture. In particular, 10.0 g of THMA (57.1 mmol) and 2.0 g TCMC (thiols substitution degree: 3.73%) were dissolved in 100 mL of deionized water. After mixing well, 200 mg of Gel@Cur nanoparticles were added to the above solution. Subsequently, 200 mg of PEGDA and 200 mg of I2959 (890 μmol) were added to form the hydrogel precursor solution. The concentration of each component of the mixture was THMA (10 wt%), TCMC (2 wt%), Gel@Cur (0.2 wt%), PEGDA (0.2 wt%) and I2959 (0.2 wt%). The precursor solution was added to a hydrogel mould and irradiated with UV light for 5 min. The resulting gel was named as THMA/TCMC‐Gel@Cur hydrogel. The hydrogel column was lyophilized, brittle fractured using liquid nitrogen and the pore structure of the hydrogel cross section was observed by SEM. The experimental procedures for SEM of Gel@Cur, synthesis and characterization of TCMC and mechanical and swelling tests of hydrogels are described in the Supplementary material.

### 2.3 In vitro antimicrobial and free radical scavenging of hydrogels

The in vitro antimicrobial capacity of hydrogels was evaluated using *Staphylococcus aureus* (*S. aureus*, San Yao Science & Technology Co., Ltd., Beijing, China) and *Escherichia coli* (*E. coli*, San Yao Science & Technology Co., Ltd., Beijing, China). The various components were co‐cultured in 2 mL of the same concentration of bacterial suspension for 8 h. The co‐cultured bacterial suspension of each group was diluted 10^8^‐fold and spread on agar plates, which were then maintained at 37°C for 12 h. The number of colonies on the agar plates of each group was recorded. To gain a more comprehensive understanding of the antibacterial properties of each component material, each component was co‐cultured with the bacterial suspension for 48 h. The absorbance at 620 nm of the co‐cultured bacterial suspension was measured and recorded within 48 h using an enzyme marker (PlateDirect A96, USA).^19^

Two different kits, the DPPH radical scavenging kit (Solarbio, Beijing, China) and the ABTS radical scavenging kit (Solarbio, Beijing, China), were used to evaluate the in vitro antioxidant activity. First, 50 mg of the sample was extracted with 1 mL of the extraction solution. The DPPH experiments were performed according to the manufacturer's instructions. First, the DPPH working solution was prepared before the experiment. Taking the sample tube as an example, each tube should contain 25 μL of sample and 975 μL of DPPH working solution. The detection wavelength was 517 nm, and the DPPH clearance ratio (DCR) was calculated using Equation (1)^20^:

(1) $$\mathrm{DCR}\left(\%\right)=\frac{A_{b}-\left(A_{s}-A_{c}\right)}{A_{b}}\times 100\%$$

In this experiment, the absorbance of the blank group (*A*_b_) was measured, as well as that of the sample group (*A*_*s*_) and the sample control (*A*_*c*_). The test was repeated three times to ensure the reliability of the results.

The ABTS experiments were performed according to the manufacturer's instructions. First, the reagent IV working solution and ABTS working solution were prepared before the experiment. Taking the sample tube as an example, each tube should contain 10 μL of sample, 20 μL of Reagent IV working solution, and 170 μL of ABTS working solution. And the absorbance was measured at 734 nm. The ABTS clearance ratio (ACR) was calculated using Equation (2)^18^:

(2) $$\mathrm{ACR}\left(\%\right)=\frac{A_{b}-\left(A_{s}-A_{c}\right)}{A_{b}}\times 100\%$$

In this experiment, the absorbance of the blank group (*A*_b_) is used as a reference point, while the absorbance of the sample group (*A*_*s*_) and the sample control (*A*_*c*_) are used to assess the impact of the experimental variables.

### 2.4 In vitro drug release and blood compatibility of hydrogels

The THMA/TCMC‐Gel@Cur (0.2 g) was mixed with PBS (10 mL) and supplemented with another PBS containing 0.2 mM H_2_O_2_ (10 mL). The PBS extract (4 mL) was then prepared for absorbance measurement at a wavelength of 425 nm, which was used to evaluate the drug release efficiency.

Four commonly used kinetic models were used to fit the in vitro drug release data from the hydrogel to gain further insight into the mode of release of Cur from the composite hydrogel: the zeroth‐order model (Equation (3)), the first‐order model (Equation (4)), the Higuchi model (Equation (5)), and the Korsmeyer–Peppas model (Equation (6)).^21^, ^22^

(3) $$Q=k_{0}t$$

(4) $$\mathrm{Ln}Q=\mathrm{Ln}Q_{0}-k_{1}t$$

(5) $$Q=k_{H}t^{\frac{1}{2}}$$

(6) $$\mathrm{Ln}Q=n\mathrm{Ln}t+{\mathrm{Ln}k}_{p}$$

In the context of this study, the variables (*Q*), (*k*_0_), (*k*_1_), (*n*), and (*k*_H_) represent cumulative percent Cur release and rate constants for the zero order, the first order, the Higuchi model, and the Korsmeyer–Peppas model, respectively.

### 2.5 Cell viability review of hydrogel

The hydrogel cytocompatibility was analyzed using human umbilical vein endothelial cells (HUVECs, Newgainbio Biological Co., Ltd., Wuxi, China). Hydrogels (diameter 15 mm, height 2 mm) were subjected to UV light exposure, following which they were soaked in DMEM medium under identical conditions for a further 48 h. HUVECs were seeded into 96‐well plates, filled with DMEM to a density of 4000 cells per well. Subsequently, the cells underwent incubation for a period of 48 h. Cell viability evaluation was conducted by quantifying the absorbance at a wavelength of 450 nm using the CCK‐8 assay kit (Jianjian Technology Co., Ltd., Nanjing, China). Cell viability was analyzed by staining live and dead cells with acridine orange/ethidium bromide (AO/EB, Yeasen Technology Co., Ltd., Shanghai, China) followed by observation by inverted fluorescence microscopy.

### 2.6 Infected wound model and treatment

The study was approved by the Animal Ethics Committee of Guangzhou Medical University (Acceptance number: G2024‐312). All animals were treated in strict compliance with the National Research Council Guide for the Care and Use of Experimental Animals. In this study, 36 male rats (weighing 200–250 g) from the Laboratory Animal Center, Guangzhou Medical University, Guangzhou City, China, were employed to investigate the wound healing properties of hydrogels. The rats were assigned 6 rats per group, and anesthetized with sodium pentobarbital (30 mg/kg), and a 10 mm puncture tool was used to create a circular wound (diameter: 10 mm) on the dorsum. The wound was then swabbed with a sterile swab soaked in *S. aureus* solution (10^6^ CFU/mL). After the establishment of infected wound models, hydrogel was applied over the wounds and fixed with medical bandages. Rats were euthanized on day 14 after wounding.

### 2.7 Statistical analysis

All in vitro and in vivo data represent at least three independent experiments. All of the data in this study were analyzed using mean values, and statistical tests such as *t*‐tests and one‐way analysis of variance were carried out using the SPSS software. Significance was indicated by the following markers: * for *p* ≤0.05, ** for *p* ≤0.01, and *** for *p* ≤0.001.

## 3 RESULTS AND DISCUSSION

### 3.1 Preparation of THMA/TCMC‐Gel@Cur hydrogel

**FIGURE 2:**
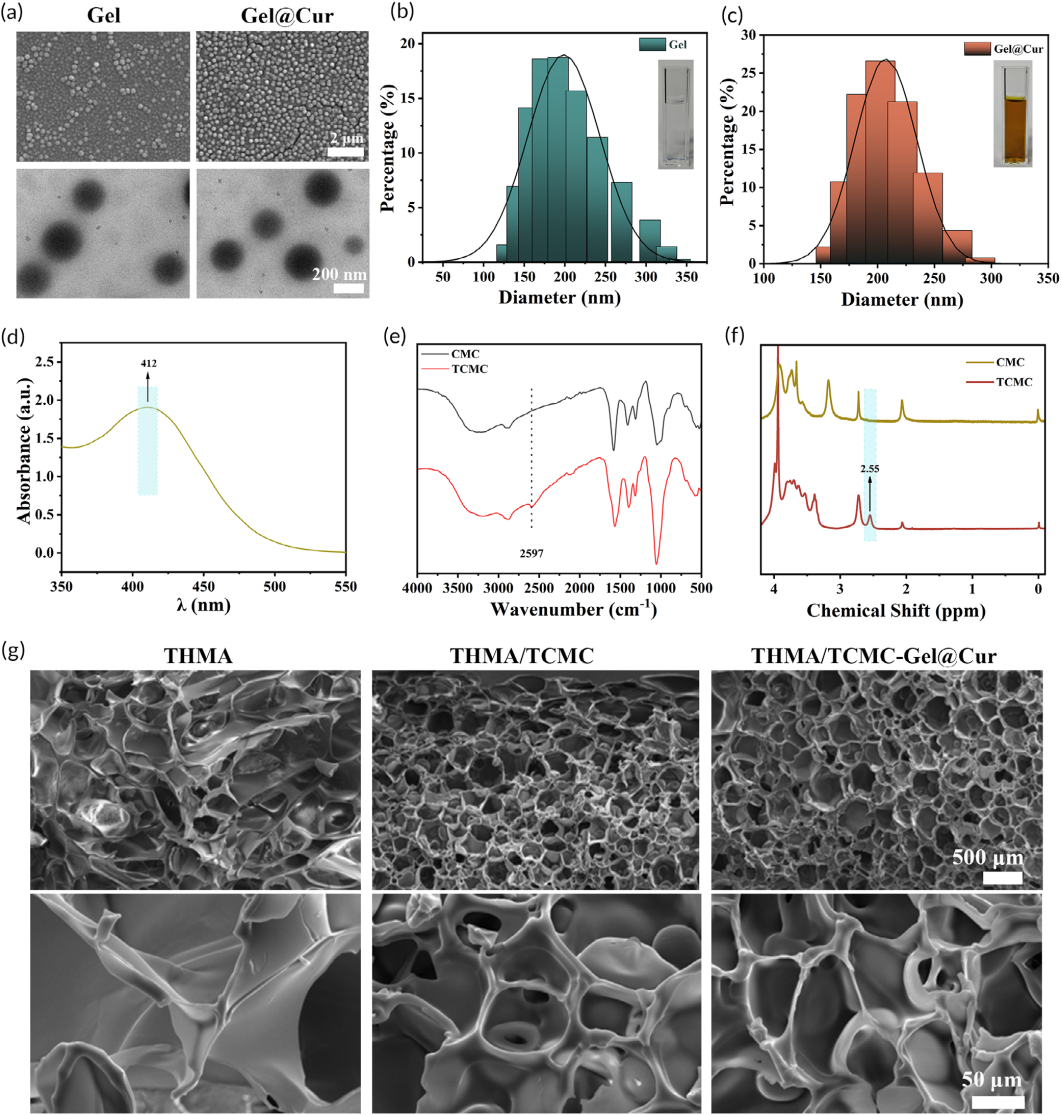
(a) The SEM and TEM images of Gel and Gel@Cur. (b), (c) Size distribution histogram of gel and Gel@Cur nanoparticles. (d) UV–vis absorbance spectroscopy in Ellman assay. (e) CMC and TCMC FTIR spectra. (f) ^1^H‐NMR spectra of CMC and TCMC. (g) SEM images of THMA, THMA/TCMC and THMA/TCMC‐Gel@Cur.

The SEM and TEM images of Gel and Gel@Cur were shown in Figure 2a, respectively. The images showed that there were no significant differences in morphology between Gel@Cur nanoparticles and Gel nanoparticles. The particle size distribution histograms of Gel and Gel@Cur were shown in Figure 2b,c, respectively. This phenomenon was attributed to the hydrophobic interaction between the hydrophobic Cur molecule and the hydrophobic region of the gelatin molecule, resulting in a more uniform size distribution of the prepared Gel@Cur nanoparticles.^23^, ^24^ The images in the right side of Figure 2b,c demonstrated a notable color difference between Gel@Cur nanoparticles and Gel nanoparticles, which confirmed the successful preparation of Gel@Cur nanoparticles.

The preparation of TCMC was conducted in accordance with the methodology described in the literature.^25^ The result of Ellman assays suggested that the thiol content of TCMC was 17.06 μmol. The degree of NH_2_ in CMC (Upon completion of the amidation reaction, the NH_2_ is successfully substituted with SH) was calculated to be 3.73% (Figures 2d and S1). The successful preparation of TCMC was also confirmed through the utilization of FTIR and ^1^H‐NMR analyses. As illustrated in Figure 2e, the peak at 2597 cm^−1^ could be assigned to SH in TCMC as described in Reference [26]. To further substantiate the successful preparation of TCMC, the distinction between CMC and TCMC was investigated through ^1^H‐NMR analysis, the results of which were presented in Figure 2f. The peaks at 2.55 ppm corresponded to the resonance of methylene protons linked to thiol groups (–CH_2_–SH).^27^ These changed peaks indicated that TCMC has been successfully synthesized.

The cross‐sectional SEM images of the prepared hydrogels were presented in Figures 2g and S2. It could be observed that the gel network became denser following the addition of TCMC, which was attributed to the generation of additional thioether bonds between the thiols and the double bonds due to the click chemistry, resulting in a denser gel network.^28^ The inclusion of the Gel@Cur led to an increase in gel pore size, indicating that the nanoparticles influenced the cross‐linking of the hydrogel network through the spatial site resistance effect.

### 3.2 Mechanical and swelling tests of hydrogels

**FIGURE 3:**
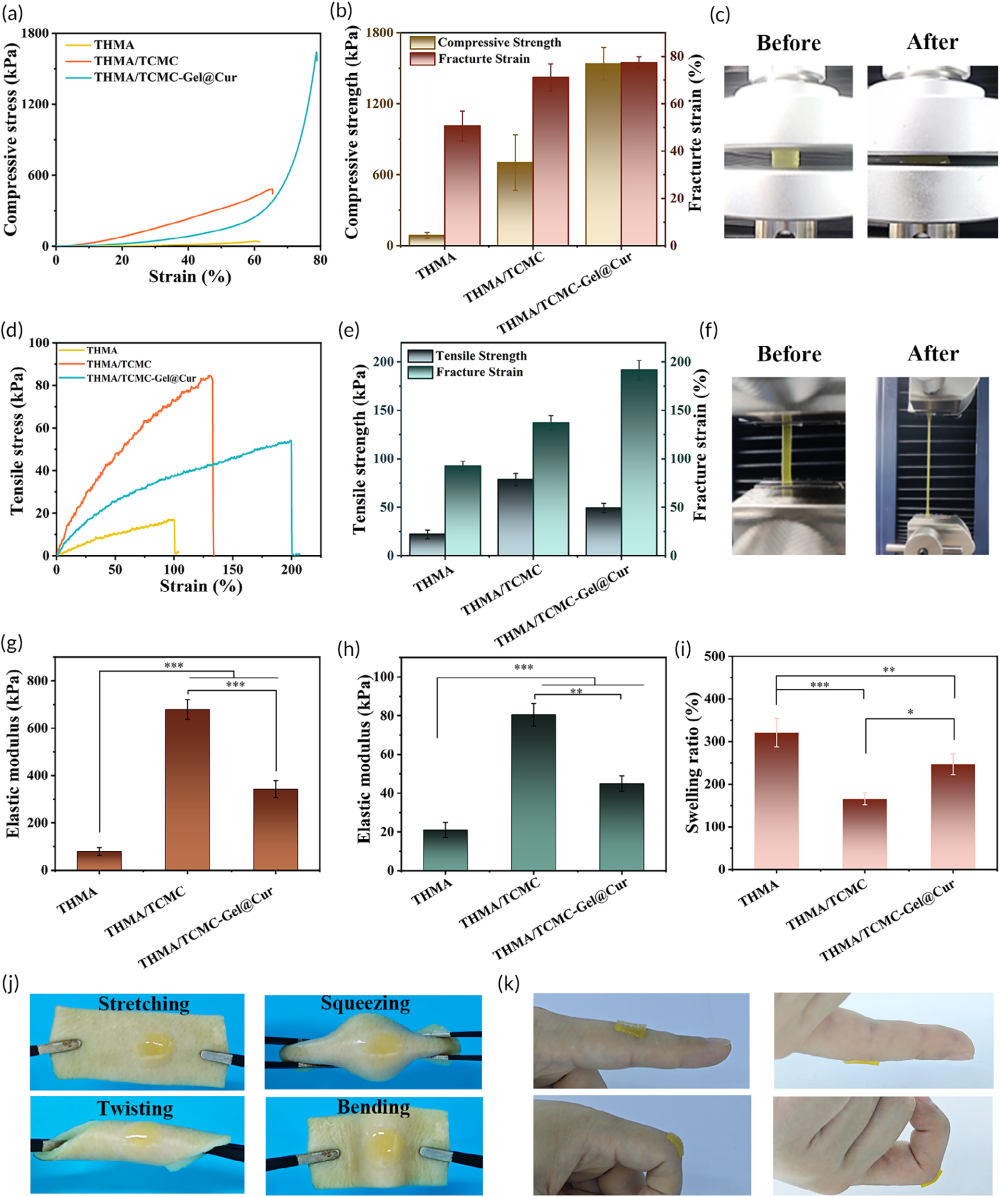
(a) Hydrogel compressive stress–strain curve. (b) Fracture strain and fracture stress of hydrogels under compression. (c) Before and after compression images of THMA/TCMC‐Gel@Cur hydrogel. (d) Hydrogel tensile stress–strain curve. (e) Breaking strain and breaking stress for hydrogels. (f) Before and after photographs of THMA/TCMC‐Gel@Cur hydrogel stretching. (g) Compressive modulus of elasticity of hydrogels. (h) Tensile modulus of elasticity of hydrogels. (i) Swelling ratio of hydrogels. (j) THMA/TCMC‐Gel@Cur hydrogel after stretching, twisting, squeezing and bending in porcine skin. (k) THMA/TCMC‐Gel@Cur hydrogel after bending in human skin.

The compressive stress–strain curves for each group of hydrogels and the compressive fracture stress and fracture strain for each group of hydrogels were presented in Figure 3a,b. The data showed that the compression performance was improved by the addition of TCMC, which was linked to the addition of additional thioether bonds within the gel, resulting in the improved compression resistance of the THMA/TCMC hydrogels.^29^ A photographic comparison of the gel before and after compression was shown in Figure 3c. At 60% strain, the THMA/TCMC‐Gel@Cur hydrogel remained unbroken. Figure 3d,e showed the tensile stress–strain curves for each group of hydrogels and the tensile rupture stress and rupture strain for each group. The data showed that the tensile strength of the THMA/TCMC‐Gel@Cur hydrogel was increased by the addition of Gel@Cur. As shown in Figure 3f, the THMA/TCMC‐Gel@Cur hydrogel remained unbroken at 200% strain. To further understand the effect of TCMC and Gel@Cur nanoparticle incorporation on composite hydrogel mechanical properties, the mechanical properties were determined for each group of gels using the elastic modulus. As shown in Figure 3g,h, the elastic modulus increased with the addition of TCMC, both in compression and in tension, which was due to the addition of thioether linkages within the gels. On the other hand, the elastic modulus of the hydrogels decreased after the addition of Gel@Cur, which was attributed to steric hindrance by the added Gel@Cur on cross‐linking of the hydrogel network.

Testing of the swelling characteristics of hydrogels was carried out and the results were shown in Figure 3i. The figure showed that with the addition of TCMC to form additional cross‐links, the density of the network fibers in the gel increases, and the swelling ratio of the composite hydrogels gradually decreases. By incorporating Gel@Cur, which had the potential to influence the cross‐linking of the gel network due to the steric hindrance effect, there was a consequent decrease in the network fiber density within the gel and a corresponding increase in the swelling ratio. This was consistent with the results of scanning electron microscopy images and mechanical analyses. Additionally, twisting, bending, and stretching tests confirmed the tight adhesion of THMA/TCMC‐Gel@Cur hydrogel to the skin (Figure 3j,k). Therefore, THMA/TCMC‐Gel@Cur hydrogel exhibited skin adhesion and adapted to the movement of skin tissues.

### 3.3 In vitro antimicrobial and free radical scavenging of hydrogels

**FIGURE 4:**
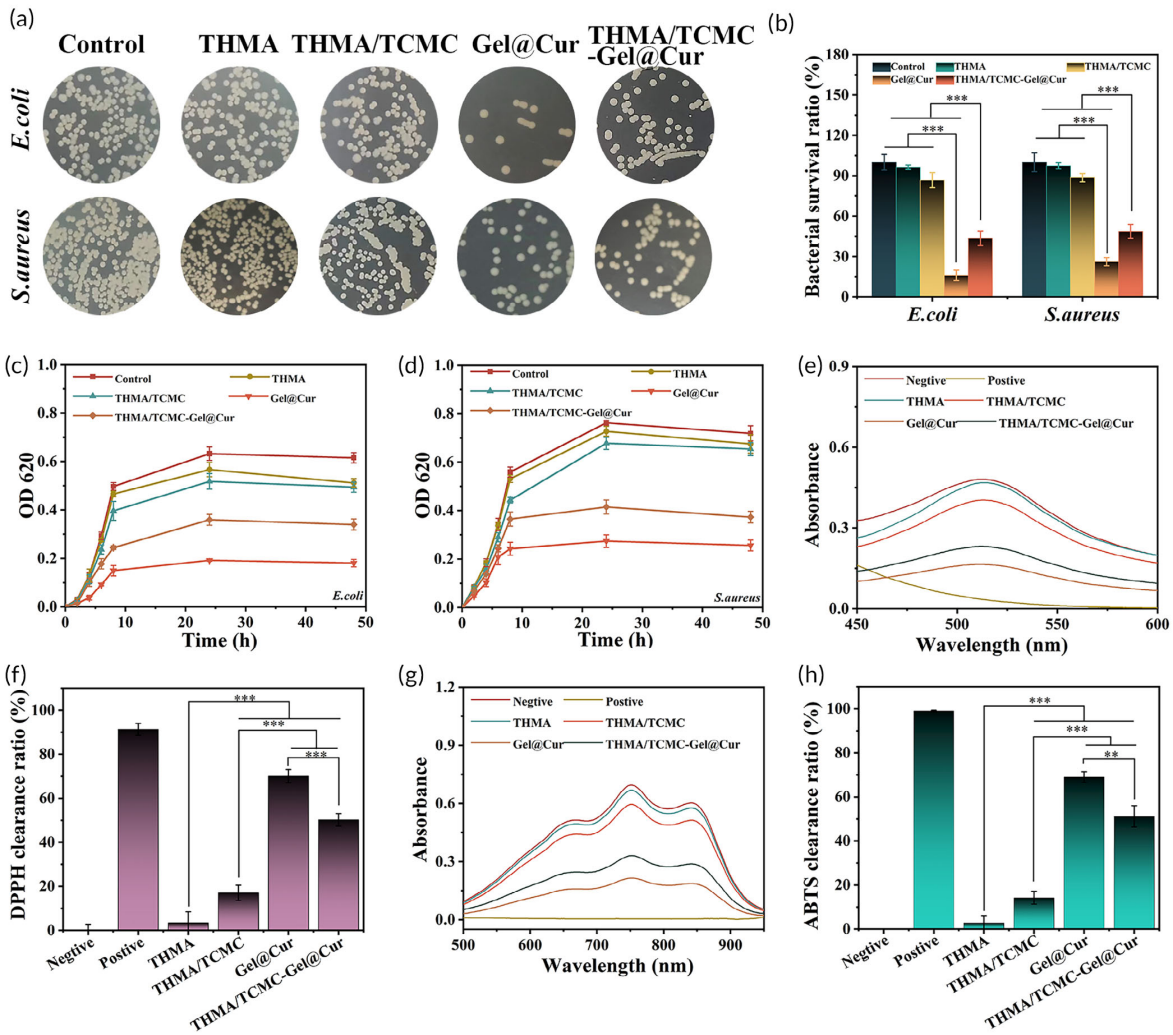
(a) Pictures of *Escherichia coli* and *Staphylococcus aureus* colonies after 8 h of co‐culture on materials. (b) Survival of *E. coli* and *S. aureus* colonies after 8 h of co‐culture on materials. (c) Curve showing the growth of *E. coli* after 48 h of co‐culture with materials. (d) *S. aureus* growth curve during 48 h of co‐culture with materials. (e) In vitro DPPH radical scavenging curves of materials. (f) DPPH clearance ratio of materials in vitro. (g) ABTS radical scavenging curves of materials in vitro. (h) ABTS in vitro clearance ratio of materials.

Excellent antibacterial properties are essential for the treatment of infectious wounds with hydrogel dressings. The bacterial colony photographs of the co‐culture of hydrogel and nanoparticles for 8 h were presented in Figure 4a. It could be observed from the figure that the number of bacterial colonies co‐cultured with THMA and THMA/TCMC hydrogels showed little difference from that of the Control group, while the number of bacterial colonies co‐cultured with THMA/TCMC‐Gel@Cur hydrogel was significantly less than that of the Control group. The release of Cur from the THMA/TCMC‐Gel@Cur required its initial diffusion from the gelatin matrix, followed by release through the hydrogel network. Consequently, the antibacterial efficacy of the THMA/TCMC‐Gel@Cur was lower than that of Gel@Cur under equivalent testing conditions. The rapid Cur release of Gel@Cur led to suboptimal bioavailability. To address this, the incorporation of Gel@Cur into the THMA/TCMC hydrogel is designed to prolong its therapeutic duration and maintain stable local drug concentrations, which is particularly critical for the long‐term treatment of chronic infectious wounds. Quantitative analysis (Figure 4b) revealed that the bacterial survival ratio co‐cultured with THMA/TCMC‐Gel@Cur hydrogel was 43.47 ± 5.35% (*S. aureus*) and 48.64 ± 5.21% (*E. coli*), respectively, demonstrating a significant antibacterial effect over the Control group. This was due to using Gel@Cur, which was able to release Cur molecules that could interact with the FtaZ protein essential for bacterial division, thereby inhibiting the growth and reproduction of bacteria.^30^ To further manifest the long‐term antibacterial characteristics of the hydrogels, the hydrogels were co‐cultured with *S. aureus* and *E. coli* for 48 h, and the bacterial growth curves were plotted (Figure 4c,d). It could be observed from the figures that the THMA/TCMC‐Gel@Cur hydrogel could sustainably inhibit bacterial growth within 48 h. This was attributed to the dense structure of the hydrogel prolonging the time for Cur molecules to diffuse to the outside environment, thereby endowing the THMA/TCMC‐Gel@Cur hydrogel with the property of long‐term antibacterial activity.

The infection at the wound site produces a large number of free radicals, which damage the structure and function of the cells and affect wound healing. Wound dressings with the ability to scavenge free radicals at the wound site are effective in promoting infectious wound healing.^20^ The hydroxyl radical and cation radical scavenging ability of the hydrogels in each group was tested by mixing the hydrogels with DPPH reagent and ABTS reagent. As shown in Figure 4e–h, the free radical scavenging ability of the THMA/TCMC hydrogel was improved after the addition of TCMC. The reason for this phenomenon was that TCMC was obtained by modification of chitosan, which has a certain free radical scavenging ability.^31^ In the DPPH assay, the Negative group (typically containing only DPPH working solution without any antioxidant) exhibited the highest absorbance at 517 nm, confirming the maximum presence of stable DPPH radicals in the initial system. In contrast, the Positive group (ascorbic acid) showed the lowest absorption. And both the THMA and TCMC‐Gel@Cur groups showed a significantly lower absorbance at this wavelength. This decrease indicated TCMC‐Gel@Cur had DPPH radical scavenging ability. Similarly, TCMC‐Gel@Cur groups demonstrated comparable trends in the ABTS assay, with reduced absorbance relative to the Negative group, confirming the free radical scavenging ability of TCMC‐Gel@Cur. Notably, the free radical clearance ratio of the THMA/TCMC‐Gel@Cur hydrogel obtained by adding Gel@Cur reached 50.24% ± 2.78% (DPPH radical) and 51.19% ± 4.81% (ABTS radical). This was attributed to the Cur released by Gel@Cur, where the phenolic ketone and methylene groups in the Cur molecule were able to form a stable structure with the free radical, thus effectively scavenging the free radical.^32^ In summary, the excellent free radical scavenging ability of the THMA/TCMC‐Gel@Cur hydrogel rendered it a potential candidate product for clinical application in infectious wound healing.

### 3.4 In vitro drug release and the regulation of hydrophilicity and hydrophobicity

**FIGURE 5:**
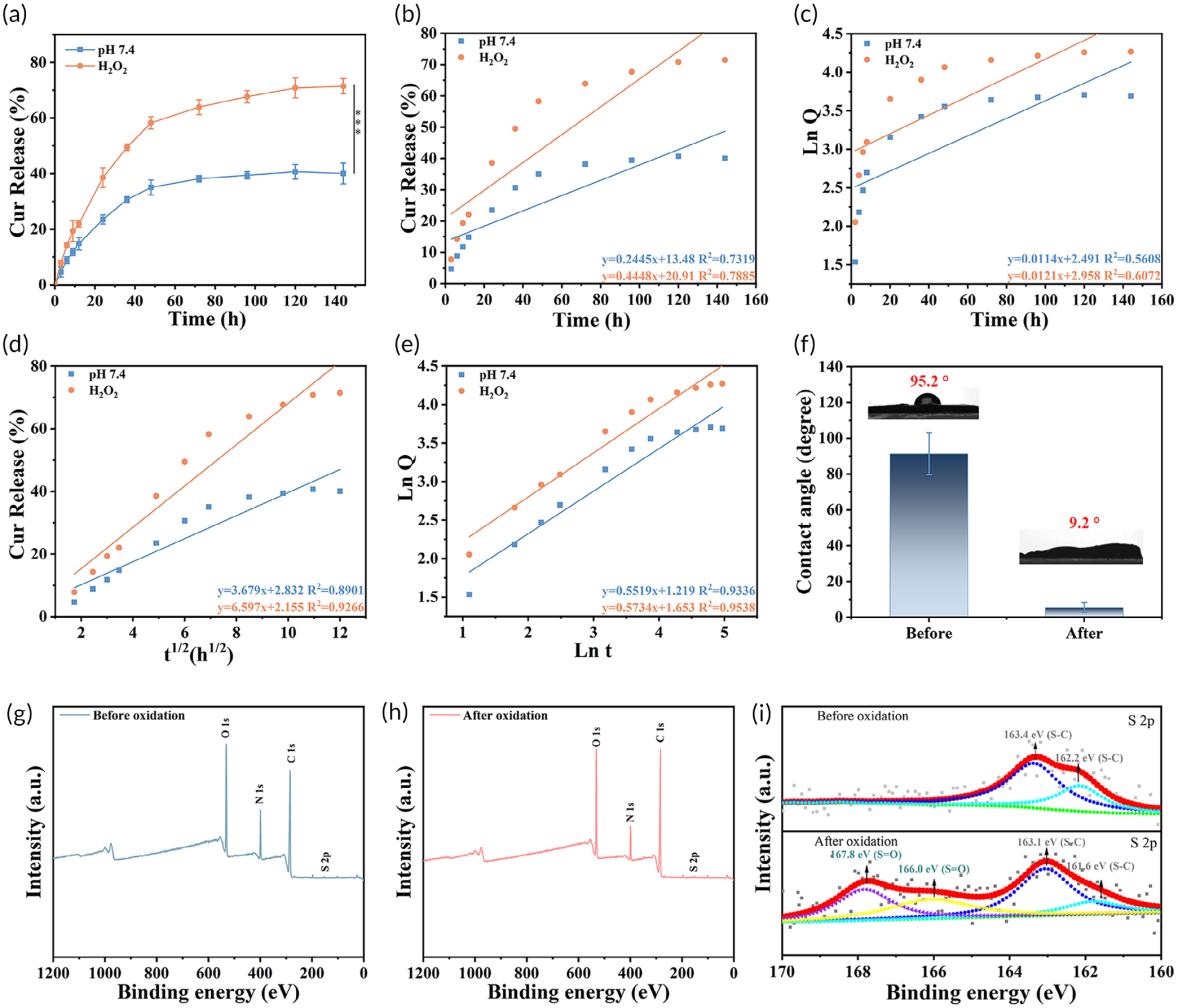
(a) Drug release profiles of THMA/TCMC‐Gel@Cur hydrogel in different liquid environments. (b) Zero‐order model fitted to hydrogel drug release results. (c) First‐order model fitted to hydrogel drug release results. (d) Higuchi model fitted to hydrogel drug release results. (e) Korsmeyer–Peppas model fitted to hydrogel drug release results. (f) Water contact angles of THMA/TCMC‐Gel@Cur hydrogel before and after 0.02 mmol/L H_2_O_2_ treatment. (g), (h). XPS pattern of THMA/TCMC‐Gel@Cur hydrogel before and after oxidation. (i) Fine XPS spectrum of S2p in THMA/TCMC‐Gel@Cur hydrogel before and after oxidation.

The direct application of Cur in wound treatment is limited by its poor water solubility and short serum half‐life. Literature has shown that controlling the release time of hydrophobic drugs can maintain the serum concentration of drugs in the affected area to promote wound healing.^33^ The drug release profiles of THMA/TCMC‐Gel@Cur hydrogel in different liquid environments were shown in Figure 5a. The addition of H_2_O_2_ to the PBS solution was able to significantly increase the drug release ratio of THMA/TCMC‐Gel@Cur hydrogel (from 40.05% ± 3.744% to 71.44% ± 2.757%). This phenomenon was attributed to the ROS‐responsive property of the thioether bond within the hydrogel. When the thioether bond is exposed to ROS, the thioether bond is oxidized to form a hydrophilic sulfoxide or sulfone. This oxidation process promotes drug release.^5^ Besides this, the addition of H_2_O_2_ may also attack the chitosan backbone, leading to oxidative cleavage of glycosidic bonds.^34^ Therefore, the combined effects of increased polarity and reduced crosslinking density lead to accelerated drug release phenomena. The drug release process of THMA/TCMC‐Gel@Cur was fitted and analyzed using four different models to gain a better understanding of the drug release process: the zero‐order model, the first‐order model, the Higuchi model, and the Korsmeyer–Peppas model. The results of the fitting of each model were presented in Figure 5b–e. The data showed that the release of THMA/TCMC‐Gel@Cur hydrogel in both PBS solution and H_2_O_2_‐involved PBS solution environments had a better fit to the Korsmeyer–Peppas model, which indicated that the drug release process of THMA/TCMC‐Gel@Cur was non‐fick diffusion, and the drug release process was influenced by both the drug diffusion and the skeleton dissolution mechanism simultaneously. Supplementary Video 1 showed the hydrophilicity–hydrophobicity transition of the hydrogel before and after H_2_O_2_ treatment. The THMA/TCMC‐Gel@Cur hydrogel surface is hydrophobic initially, causing water droplets to maintain a spherical shape. Following H_2_O_2_ treatment, the droplets in the surface obviously spread, forming a thin film with a near‐zero contact angle. In addition, the effect of ROS on the drug release of the hydrogel was confirmed by contact angle measurement. As shown in Figure 5f, the water contact angle measurement showed that THMA/TCMC‐Gel@Cur hydrogel exhibited surface hydrophobicity with a water contact angle of 95.2°. However, it became hydrophilic after the surface was treated with H_2_O_2_, with water contact angles following treatment close to 0°. This meant that hydrophobic thioether bonds could be oxidized to hydrophilic sulfoxide bonds by ROS. XPS pattern demonstrated the conversion of thioether bonds into sulfoxide bonds. The peaks at 162.2 and 163.4 eV were associated with the thioether bond. Upon oxidation, a signal emerged at approximately 167.8 eV, corroborating the formation of the S=O bond (Figure 5g–i). Therefore, the surface of THMA/TCMC‐Gel@Cur hydrogel was readily wetted by water after H_2_O_2_ treatment, promoting rapid infiltration of moisture into the interior, enlarging the pores, and accelerating drug diffusion, typically resulting in an increased drug release rate.

### 3.5 Assessment of cytotoxicity, cell spreading and cell migration

**FIGURE 6:**
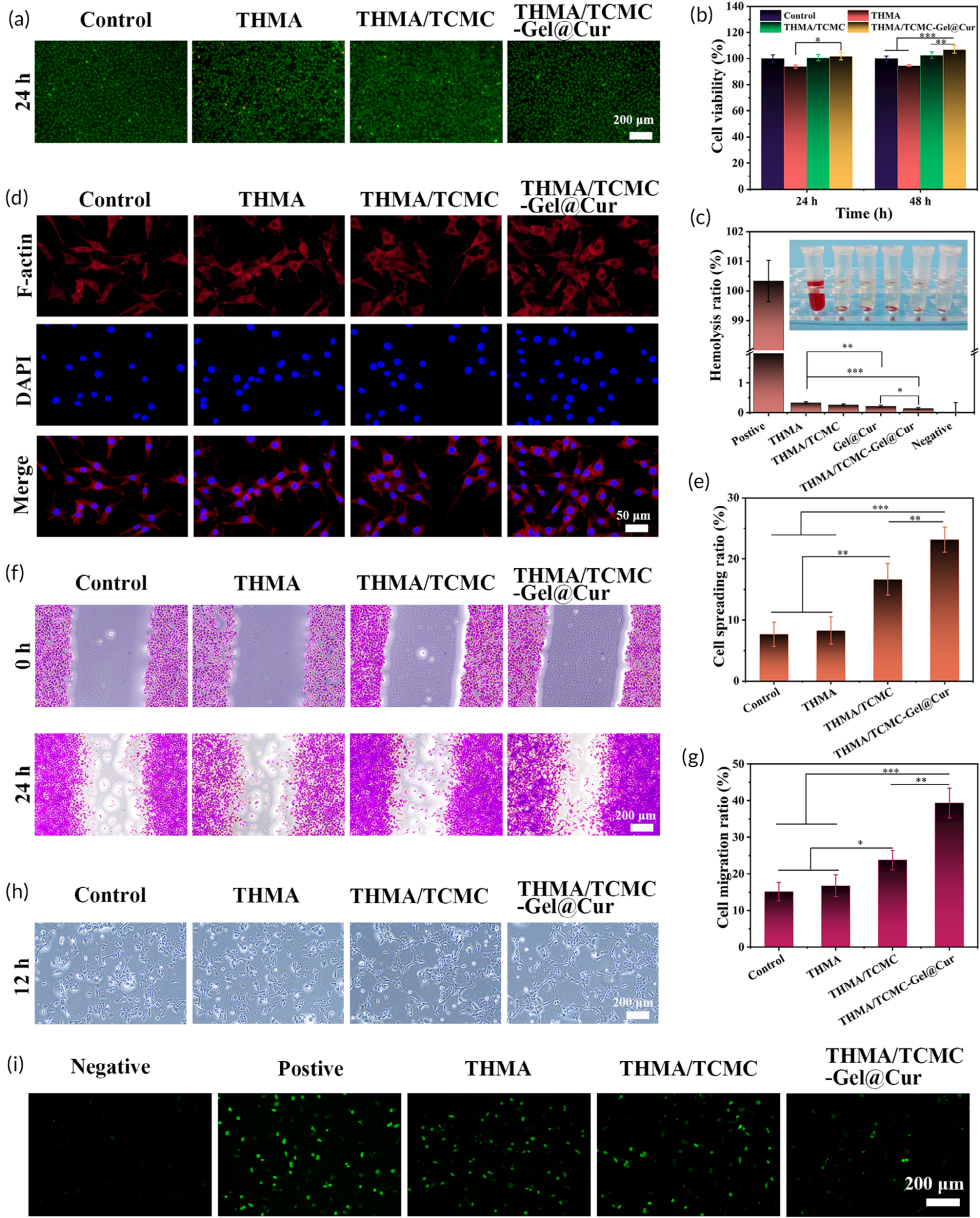
(a) HUVECs cultured with material extract for live/dead cell staining. (b) CCK8 assay for HUVECs viability. (c) Hydrogels and nanoparticle hemolysis ratio. (d) Image of the HUVECs cytoskeleton. (e) Cell spreading ratio quantification after treatment with the material extract. (f) Migration images of HUVECs treated with the material extract. (g) Quantification of HUVECs mobility under treatment with the material extract. (h) Photographs of cell vascularization treated with the material extract. (i) Photographs of intracellular ROS fluorescence in vitro.

The cytotoxicity of the hydrogels and nanoparticles was tested using a leaching method. As could be seen in Figures 6a,b and S3, cell number increased significantly and cell viability was higher than 90% in each hydrogel group, indicating good cell growth throughout the experiment. It was worth noting that the cells cultured in the THMA/TCMC‐Gel@Cur hydrogel group were significantly different from the other hydrogel groups after 48 h of culture, and this phenomenon was due to the addition of TCMC and Gel@Cur nanoparticles. TCMC was modified by chitosan with excellent biocompatibility, and gelatin had excellent biocompatibility. Therefore, the THMA/TCMC‐Gel@Cur hydrogel was not cytotoxic.

It was essential that skin repair dressings demonstrated good blood compatibility to be suitable for use. The blood compatibility of the materials was evaluated through a hemolytic activity test. The appearance and color of the solutions in the hydrogels and nanoparticle groups were observed and recorded (Figure 6c). All material groups exhibited pale yellow coloration, consistent with the saline group (negative), while the deionized water group (positive) displayed a bright red hue. The quantitative results demonstrated that the hemolysis ratio of all materials complied with the international standard for the testing of medical materials, ISO 10993‐4:2017 (hemolysis ratio less than 5%).^29^ It is noteworthy that the THMA/TCMC‐Gel@Cur hydrogel exhibited the lowest hemolysis ratio (0.1392% ± 0.0313%), thereby demonstrating that the hydrogel has favorable blood compatibility properties.

To assess the effect of the hydrogel on the morphology of HUVECs, phalloidin (red) and DAPI (blue) solutions were used in this study to stain the actin and nuclei of HUVECs. The staining results were shown in Figure 6d. Filamentous actin was increased in the cells of HUVECs treated with THMA/TCMC‐Gel@Cur hydrogel compared with the control group and other hydrogel groups. Based on the data analysis shown in Figure 6e, it was clear that filamentous actin was significantly increased in the hydrogel‐treated HUVECs in the THMA/TCMC‐Gel@Cur group, which was attributed to the effect of the gelatin within the Gel@Cur nanoparticles on the HUVECs, and thus the THMA/TCMC‐Gel@Cur hydrogel promoted vascular endothelial cell extension.

The migration and proliferation abilities of HUVECs with different hydrogel treatments were assessed by scratch experiments. As shown in Figure 6f,g, after incubation with the material extracts for 24 h, there was a significant difference in cell migration ratio between the THMA/TCMC and the THMA group, which was attributed to the increased biocompatibility of the hydrogel due to the participation of TCMC. There was a significant difference in cell migration ratio between the THMA/TCMC‐Gel@Cur group and the other groups. This was attributed to the incorporation of Gel@Cur nanoparticles, as gelatin had the ability to induce cell adhesion and migration, and thus the THMA/TCMC‐Gel@Cur hydrogel could enhance the migration and spreading of HUVECs.

### 3.6 In vitro angiogenesis and ROS scavenging assessment

The impact of the hydrogel on the angiogenic potential of HUVECs was evaluated through an in vitro angiogenesis assay. The experimental images and quantitative data were presented in Figure 6h. After 12 h of incubation, the vessel length and number of lumens were significantly higher in the THMA/TCMC‐Gel@Cur group than in the other groups. This indicated that the THMA/TCMC‐Gel@Cur hydrogel possessed a significant angiogenic effect. It was achieved by adding Gel@Cur nanoparticles, in which gelatin molecules within the Gel@Cur nanoparticles existed RGD sequences (Arg‐Gly‐Asp), which were able to promote the adhesion, proliferation, and angiogenesis of HUVECs.^35^

In vitro ROS scavenging assay was employed to provide further insight into the effect of Gel@Cur nanoparticle incorporation on HUVECs subjected to oxidative stress. The results in Figure 6i irrefutably demonstrated that THMA/TCMC‐Gel@Cur hydrogel was efficacious in scavenging ROS from cells. The observed effect was attributed to the presence of Cur within the Gel@Cur nanoparticles, which were composed of Cur molecules that had been demonstrated to mitigate intracellular ROS levels and consequently alleviate cellular oxidative stress. The aforementioned results indicated that THMA/TCMC‐Gel@Cur hydrogel possessed favorable cytocompatibility and had potential clinical applications.

### 3.7 Infected wound model for in vivo wound repair

**FIGURE 7:**
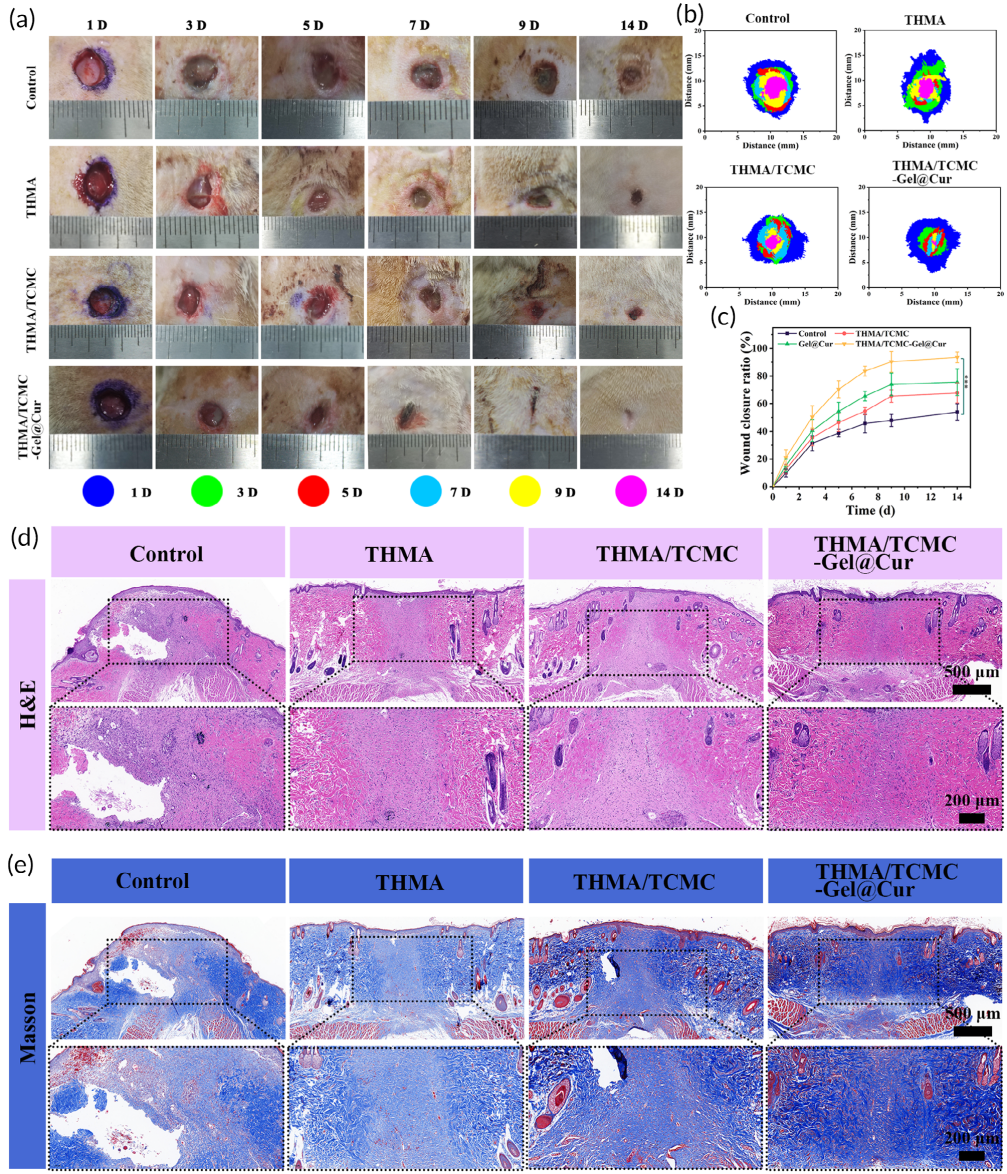
(a) Images of the injured sites of rats after 14 days in different treatment conditions. (b) Superimposition of wounds of rats after 14 days in different treatment conditions. (c) Curves of the wound closure ratio in rats after 14 days in different conditions of treatment. (d) H&E‐stained images of wound site skin tissue from rats on 7 and 14 days under different treatment conditions. (e) Images of Masson's staining of rat skin tissue at the wound site on 7 and 14 days.

An infected wound rat model was used to evaluate the therapeutic efficacy of different hydrogels on infected wounds. Photographs of the wounds on the back of the rats and the quantitative wound closure curves over the 14 days were shown in Figure 7a–c. Each group demonstrated a significantly greater ability to promote wound closure than control, due to its dense texture, physically isolating external bacteria and keeping the wound moist to promote cell growth and migration. Although Gel@Cur exhibited rapid onset of action, Gel@Cur in the wound environment was susceptible to rapid clearance by wound exudate or diffusion into surrounding tissues, leading to a sharp decline in effective local concentration and compromised long‐term efficacy. To overcome this limitation, Gel@Cur nanoparticles were loaded into the hydrogel. It was noteworthy that the hydrogel treatment of wound closure was significantly faster in the THMA/TCMC‐Gel@Cur group than in the THMA and THMA/TCMC groups. On the 14th day, the wound closure ratio of THMA was only 67.78% ± 7.27%, while the wound closure ratio of THMA/TCMC‐Gel@Cur hydrogel was 93.56% ± 3.96%. This had been attributed to the sustained release of Cur in THMA/TCMC Gel@Cur, which not only inhibited bacterial ingress but also reduced cellular oxidative stress to accelerate wound healing. The hydrogels in the THMA/TCMC‐Gel@Cur group showed a significant ability to promote wound repair in infected skin.

The histopathological structure of the injured skin was evaluated using H&E and Masson staining. New granulation tissue was present in the control wound on day 14 of skin repair, but the granulation tissue was infiltrated by a large number of inflammatory cells, indicating that the inflammatory response was still present in the wound, as shown in Figure 7d. The wound area was reduced in the THMA and THMA/TCMC groups. However, there was still an inflammatory response in the wounds. The wound treated with THMA/TCMC Gel@Cur was free of inflammation, dense blood vessels and the formation of hair follicles, and the degree of epithelialization was higher than that of the control group. This trend was further demonstrated by Masson's test (Figure 7e). Collagen accumulation was significantly increased in the THMA/TCMC‐Gel@Cur group compared to the other groups, and the collagen was neatly arranged in the wounds treated with hydrogel. This finding was related to the incorporation process of Gel@Cur nanoparticles and the sustained release properties of the drug.

### 3.8 Histological observations and analysis

**FIGURE 8:**
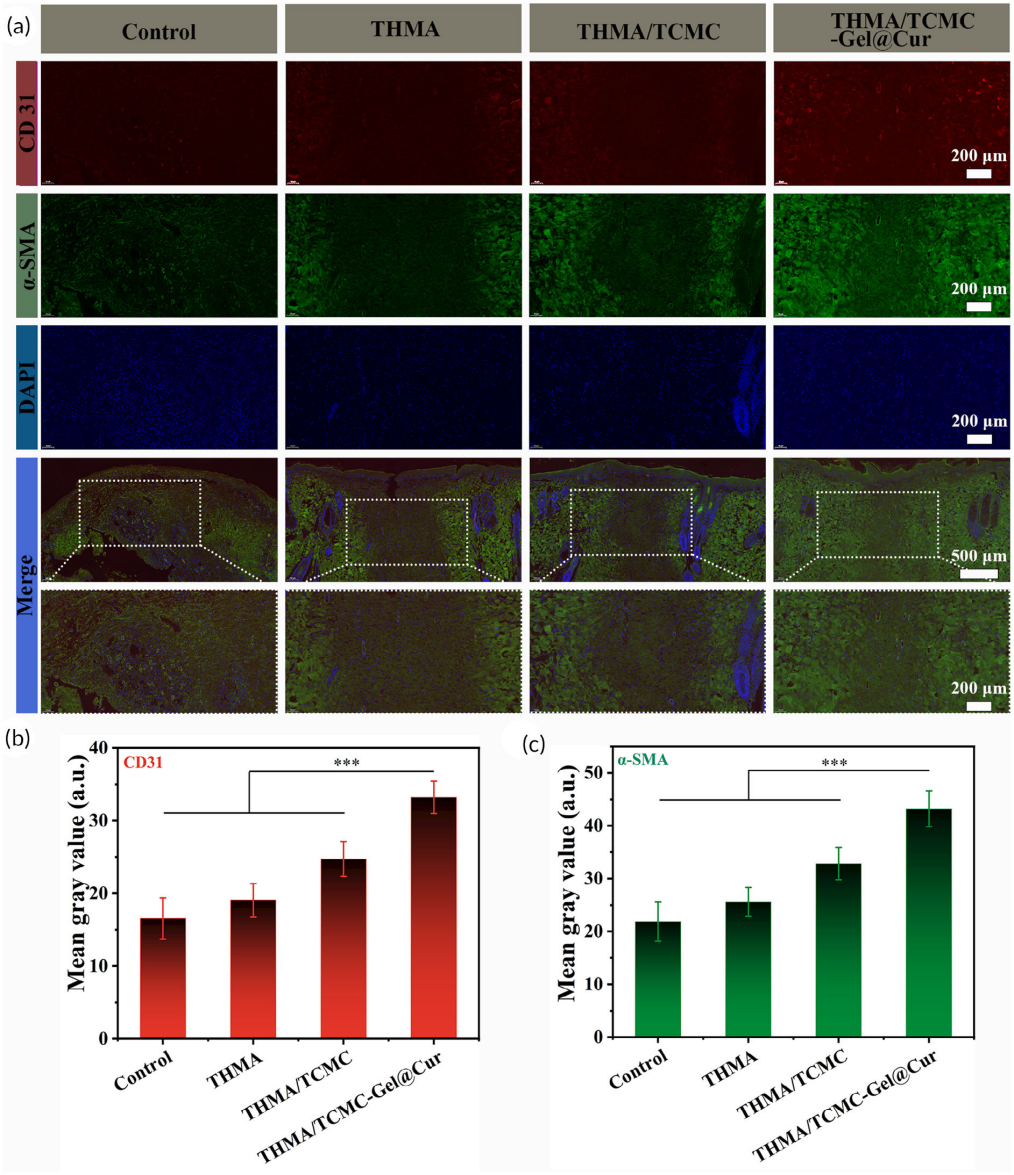
(a) Images of immunofluorescence staining in wound tissue 14 days after treatment with different materials. (b) CD31 protein absolute fluorescence intensity. (c) *α*‐SMA protein absolute fluorescence intensity.

The healing process of infected wounds treated with hydrogel was further investigated by immunofluorescence staining for CD31 and *α*‐Smooth Muscle Actin (*α*‐SMA). Fluorescent labeling of CD31 (vascular endothelial cell marker) can reflect neovascularisation at the wound site. *α*‐SMA fluorescence labeling can show the activation of myofibroblasts during wound healing. Immunofluorescence staining images and quantitative results of wound tissue were shown in Figure 8a–c. The fluorescent signals of *α*‐SMA and CD31 were stronger in the THMA/TCMC‐Gel@Cur group compared with the control, THMA and THMA/TCMC groups, indicating that the number of newly formed blood vessels and muscle fibroblasts was higher in the THMA/TCMC‐Gel@Cur treated wounds. This further suggested that THMA/TCMC‐Gel@Cur had a high wound repair capacity. Quantitative analysis of the data showed that compared to the control, THMA and THMA/TCMC groups, the THMA/TCMC Gel@Cur group had a significant increase in the number of myofibroblasts and neovascularisation after 14 days of treatment. These results highlighted the potential role of THMA/TCMC‐Gel@Cur in the promotion of wound repair in infected wounds. It had been shown that the THMA/TCMC gel@Cur could significantly accelerate the healing of wounds with infection.

## 4 CONCLUSIONS

In this study, a ROS‐responsive THMA/TCMC‐Gel@Cur composite hydrogel based on “thiol‐ene” click chemistry was developed to promote infected wound healing. The hydrogel could change the hydrophobic sulfide bond into a hydrophilic sulfoxide bond and increase the swelling ratio of the hydrogel according to the change in ROS level. It made it easier for water molecules to enter the hydrogel and accelerated the diffusion of drugs in the hydrogel through the porous network to the infected wound. The addition of Gel@Cur enhanced the ROS scavenging and in vitro antibacterial ability of the hydrogel, which effectively promoted wound healing. Animal experiments showed that the infected wound healing ratio of rats treated with THMA/TCMC‐Gel@Cur hydrogel was significantly faster. Histological observation showed that the treated wound inflammation was reduced, new angiogenesis was increased, and collagen accumulation was significantly increased. THMA/TCMC‐Gel@Cur composite hydrogel could significantly promote infected wound healing by reducing inflammation, promoting new blood vessel regeneration, and collagen deposition, showing good clinical application potential, which was expected to improve the condition of patients with infectious skin diseases and improve the quality of life.

## AUTHOR CONTRIBUTIONS

**Jinbao Zhong:** Conceptualization; methodology; writing – original draft; validation; data curation. **Bihua Liang:** Funding acquisition; software; visualization; data curation. **Shanshan Ou:** Validation; visualization; formal analysis. **Jiaoquan Chen:** Formal analysis; software. **Huaping Li:** Writing – original draft; funding acquisition; conceptualization; investigation; formal analysis. **Hui Zou:** Visualization. **Chao Bi:** Methodology; investigation; funding acquisition; validation. **Sanquan Zhang:** Writing – review and editing; resources; project administration; validation. **Huilan Zhu:** Funding acquisition; writing – review and editing; project administration; resources; supervision. **Tianyi Lin:** Supervision. **Luoyu Zhang:** Data curation; formal analysis.

## CONFLICT OF INTEREST STATEMENT

The authors declared that they have no conflicts of interest or personal relationships with this work.

## Supporting information


**Figure S1.** Standard curve in Ellman assay.
**Figure S2.** Analysis of hydrogel pore size.
**Figure S3.** HUVECs cultured with material extract in 48 h for live/dead cell staining.

**Video 1.** Demonstration of a contact‐angle experiment.

## Data Availability

The data that support the findings of this study are available from the corresponding author upon reasonable request.
