# Supplementary material for: ROS‐responsive robust hydrogels based on thiol‐ene click chemistry for enhancing the healing of infected wounds
Source: Bioeng Transl Med. 2026 Apr 8;11(4):e70143. doi: 10.1002/btm2.70143 (PMC13327624; doi:10.1002/btm2.70143)
Supplement: Supplementary file 1 — Figure S1. Standard curve in Ellman assay. Figure S2. Analysis of hydrogel pore size. Figure S3. HUVECs cultured with material extract in 48 h for live/dead cell staining. [file BTM2-11-e70143-s002.docx]

# Supplementary materials

# ROS-responsive robust hydrogels based on thiol-ene click chemistry for enhancing the healing of infected wounds

Huaping Li^†^, Jinbao Zhong^†^, Chao Bi, Bihua Liang, Shanshan Ou, Jiaoquan Chen, Luoyu Zhang, Hui Zou, Tianyi Lin, Sanquan Zhang^*^, Huilan Zhu^*^

Guangzhou Dermatology Hospital. Institute of Dermatology, Guangzhou Medical University, Guangzhou 510095, PR China.

^†^These authors contributed equally to this work and should be considered co-first authors.

^*^Corresponding authors.

E-mail: zhangsq2000@sohu.com (Sanquan Zhang), zhlhuilan@126.com (Huilan Zhu).

# 1. Materials and methods

## 1.1 Synthesis and characterization of TCMC

TCMC was synthesized according to the previously published methodology[1]. Firstly, 500 mg of CMC (56 μmol) was dispersed in a deionized water solution. Then, the solution was infused with EDC (100 mg, 520 μmol) and TGA (30 mL, 420 mmol). Subsequently, the pH was maintained at 5 using 1 M NaOH under stirring for a period of 3 h. The reaction mixture was subjected to dialysis using a 10,000 molecular weight dialysis bag, and the resulting supernatant was freeze-dried to obtain TCMC. The reaction mixture was analyzed using fourier transform infrared spectroscopy (FTIR, Thermo Fisher Nicolet IS, USA) and proton nuclear magnetic resonance spectroscopy ^(1^H-NMR, Bruker AVANCE III HD, Germany) to ascertain the differences between CMC and TCMC, thereby demonstrating the successful synthesis of TCMC.

## 1.2 Characterization of Gel@Cur nanoparticles

Gel@Cur nanoparticles were synthesized according to previously reported methods[2]. Scanning electron microscopy (SEM, Sigma300, USA) and transmission electron microscopy (TEM, JEOL F200, USA) were used to observe the morphological differences between the gel and Gel@Cur. The difference in particle diameter between Gel and Gel@Cur was tested using a particle sizer (Zetasizer Nano S90, UK)[3].

## 1.3 Ellman assay

The -SH levels were determined using the method of Ellman assay. For detailed methods please refer to Franco et al[4]. DTNB reagent was prepared by dissolving 4mg DTNB in 1 mL buffer containing 1 M sodium phosphate and 1 mM EDTA. The final reaction system contained 0.1 mg/mL DTNB working solution, sodium phosphate buffer, methanol and 100 mg/mL sample. A standard curve was generated with L-cysteine. After incubating at room temperature for 30 min, the absorbance was captured at 412 nm wavelength.

## 1.4 Mechanical and swelling tests of hydrogels

The hydrogel mechanical properties were characterized by compression and tensile tests on each group of hydrogels at 25 °C employing the universal testing machine (Norwood, USA). Before the compression test, the gels were prepared into uniform cylinders with diameters of 14.65 mm and heights of 15 mm using a mould. The compression test was performed at the specified strain rate of 2 mm/min. The fracture strain and fracture stress of each group of hydrogels were recorded for each group of samples. For the tensile property test, the hydrogels were prepared as a rectangle of 75 mm × 4 mm × 2 mm according to the mould, and the tensile test was performed at the specified strain rate of 10 mm/min to record the fracture strain and fracture stress of each group of hydrogels[5]. The elastic modulus was measured by calculating the ratio of stress to strain over the elastic deformation range of the hydrogels, and the strain range selected for both compression and tension was 20%-40%.

The swelling test was employed for the purpose of evaluating the swelling ratio (SR). The same volume of lyophilized hydrogels was placed in PBS at pH 7.4. The SR calculated from Eq. (1)[6]:

$SR(\%)=\frac{W_{s}-W_{d}}{W_{d}}\times100\%$ (1)

In this context, *W*_s_ and *W*_d_ indicate the weight of hydrogels in their swollen and dry states, respectively. The test was repeated five times.

## 1.5 Contact angle assay

A rubber ring was placed on a glass slide, and 200 µL of hydrogel precursor was pipetted into it. Following UV irradiation for 5 min, any surface free water was blotted away with filter paper. A droplet was then dispensed vertically onto the hydrogel surface, and its contact angle was measured using a contact angle goniometer. Additionally, the glass slide with the formed hydrogel was immersed in a mixture solution of 0.02 mmol/L H_2_O_2_ and PBS. After 10 s of immersion, hydrogel was removed and blotted with filter paper to eliminate excess surface water. Finally, the contact angle was measured again using the contact angle. The droplet images were captured and the corresponding contact angle values were recorded.

## 1.6 Hemolytic activity test

The hemolytic activity test was performed as previously reported. The red blood cells were collected by centrifuging the mouse blood at 3,000 rpm for 10 min. The erythrocytes were subjected to three washes with saline, after which they were diluted to a concentration of 5% (vol/vol) with saline. The hydrogel (100 µL) was mixed with the erythrocyte stock solution (900 µL) and added to the centrifuge tube, which was then shaken at 100 rpm for 1 h in a 37 °C incubator. The same volume of deionized water was used as the positive control and saline as the negative control. The contents of the centrifuge tubes were centrifuged for 10 min at 3,000 rpm and the obtained supernatant (100 µL) was transferred to 96-well plates. Solution absorbance was recorded at 540 nm using an enzyme marker (PlateDirect A96, USA). The hemolysis ratio (HR) was calculated using Eq. (8):

$HR(\%)=\frac{A_{s}-A_{n}}{A_{p}-A_{n}}\times100\%$ (2)

In this context, *A*_s_ represents the absorbance of the sample, *A*_p_ signifies the absorbance of the positive control, and *A*_n_ denotes the absorbance of the negative control.

## 1.7 Evaluation of cell migration and spreading of hydrogels

HUVECs were grown in 24-well plates at a cell density of 50000 per well. After forming a monolayer, the cells were gently scraped and rinsed with PBS, and then treated with material extracts in DMEM. The scratch closure was observed under an inverted microscope after 24 h. The area of scratches was quantified using Image J. Cell migration ratio (CMR) was calculated according to Eq. (9):

$CMR(\%)=\frac{R_{0}-R_{t}}{R_{0}}\times100\%$ (9)

In this context, *R*_0_ indicates the initial scratch area, while *R*_t_ denotes the remaining scratch area at *t*.

HUVECs were plated in a 48-well plate at 50000 cells per milliliter and maintained in DMEM medium under conditions of 37 °C with 5% CO_2_ for a duration of 24 h. Subsequently, fixation with a 4% solution of paraformaldehyde, permeabilization using 0.1% Triton X-100, and blocking with a 1% solution of BSA. Rhodamine-labelled phalloidin and DAPI solutions were used to label the cytoskeleton and nuclei, respectively. The proliferation and adhesion status were then monitored using fluorescence microscope. Cytoskeletal fluorescence intensity and area were quantitatively analyzed using ImageJ software.

## 1.8 Cell tubulation and reactive oxygen scavenging assay

Matrix gel (10µL) was evenly spread in a 96-well dish and incubated at 37 °C for 30 min to form a gel layer. HUVECs (5000 cells/well) were added to the gel layer, treated with DMEM containing material extracts and grown at 37 °C under 5% CO_2_ for 24 h. Each constituent tube was recorded and quantitatively analyzed using ImageJ.

HUVECs were seeded in 24-well plates at 20000 cells/well and cultured for 6 h at 37 °C in a 5% CO_2_ environment, and then cultured in hydrogel-soaked DMEM medium for 18 h, and then Cells were treated with medium containing H_2_O_2_ solution. After 4 h of incubation, the culture medium was removed and then exposed to the DCFH-DA probe for 30 min. ROS within the cells were examined using an inverted fluorescence microscope. A group of cells treated solely with the medium supplemented with H_2_O_2_ served as the positive control to establish the extent of oxidative stress. Conversely, a group treated with medium devoid of any H_2_O_2_ solution acted as the negative control, representing the baseline ROS levels.

## 1.9 Infected wound model and treatment

After the establishment of animal models, the rats were classified into four groups. One group served as a control group with untreated wounds, and the other three groups were treated with THMA, THMA/TCMC, and THMA/TCMC-Gel@Cur hydrogel, respectively. The sample number was six rats in each group. Throughout the healing process on days 1, 3, 5, 7, 9, and 14, the progress of wound healing in rats was monitored and recorded using a digital camera to calculate the wound healing ratio. Rats were euthanized on day 14 after wounding. Tissues were then collected from the wound site, fixed in 4% para-formaldehyde solution, embedded by paraffin and analyzed with hematoxylin and eosin (H&E) and Masson staining in order to assess tissue structure, collagen deposition and regeneration. The experiments were repeated three biological repetitions.

In order to gain a deeper understanding of the wound healing process, specific substances in tissue sections were labeled on day 7 and 14. The tissue slices were first incubated with a 10% solution of goat serum for a duration of 30 min at 25 °C. Subsequently, they were exposed to the primary antibodies for CD31 or *α*-SMA and allowed to incubate overnight under refrigerated conditions (4 °C). Following this, the segments were visualized using secondary antibodies labeled with fluorescent markers (goat anti-rabbit antibody labeled with rhodamine for CD31 and goat anti-mouse antibody labeled with FITC for *α*-SMA) at 25 °C. Nuclei were labelled by DAPI for 15 min and the presence of CD31 and *α*-SMA in immunofluorescence-labelled sections was observed by confocal laser scanning microscopy (CLSM). To determine the level of expression of CD31 and *α*-SMA, the fluorescence images obtained by CLSM were quantitatively evaluated using ImageJ software.

**Results**

**

**

Fig. S1 Standard curve in Ellman assay.

**

**

Fig. S2 Analysis of hydrogel pore size.

Fig. S3 HUVECs cultured with material extract in 48 h for live/dead cell staining.

# Reference

[1] F.G.M. Borsagli, A.J.M. de Souza and A.E. Paiva, Ecofriendly multifunctional thiolated carboxymethyl chitosan-based 3D scaffolds with luminescent properties for skin repair and theragnostic of tissue regeneration, International Journal of Biological Macromolecules, 165 (2020) 3051-3064.

[2] D. Liang, X. Shen, L. Han, H. Ren, T. Zang, L. Tan, Z. Lu, X. Liao, B.S.S. Vetha and Y. Liu, Dual‐ROS Sensitive Moieties Conjugate Inhibits Curcumin Oxidative Degradation for Colitis Precise Therapy, Advanced Healthcare Materials, 13 (2024) 2303016.

[3] M. Lan, J. Zhang, J. Zhou and H. Gu, CQDs-Cross-Linked Conductive Collagen/PAA-Based Nanocomposite Organohydrogel Coupling Flexibility with Multifunctionality for Dual-Modal Sensing of Human Motions, ACS Applied Materials & Interfaces, (2024).

[4] N. Denora, A. Lopedota, M. Perrone, V. Laquintana, R.M. Iacobazzi, A. Milella, E. Fanizza, N. Depalo, A. Cutrignelli, A. Lopalco and M. Franco, Spray-dried mucoadhesives for intravesical drug delivery using N-acetylcysteine- and glutathione-glycol chitosan conjugates, Acta Biomater, 43 (2016) 170-184.

[5] P. Nezhad-Mokhtari, H. Hamishehkar, M.R. Farahpour, A. Mehdipour, R. Rahbarghazi, M. Milani and M. Mehrali, Engineered bioadhesive Self-Healing nanocomposite hydrogel to fight infection and accelerate cutaneous wound healing, Chemical Engineering Journal, 489 (2024) 150992.

[6] F. Yang, L. Dai, K. Shi, Q. Liu, M. Pan, D. Mo, H. Deng, L. Yuan, Y. Lu and L. Pan, A facile boronophenylalanine modified polydopamine dual drug-loaded nanoparticles for enhanced anti-tumor immune response in hepatocellular carcinoma comprehensive treatment, Biomaterials, 305 (2024) 122435.
